# Single-Center Experience of Management of Mediastinal Cyst—A Case Series

**DOI:** 10.1055/s-0042-1749429

**Published:** 2022-06-30

**Authors:** Anil Gokce, Elgun Valiyev, Merve Satir Turk, Muhammed Sayan, Ali Celik, I Cuneyt Kurul, A Irfan Tastepe

**Affiliations:** 1 Department of Thoracic Surgery, Ankara City Hospital, University of Health Sciences, Bilkent, Ankara, Turkey; 2 Department of Thoracic Surgery, Gazi University Medicine Faculty, Yenimahalle, Ankara, Turkey

**Keywords:** mediastinal cysts, thymic cyst, foregut cysts, video assisted surgery

## Abstract

**Background** Mediastinal cysts are benign lesions that may be seen in adulthood as well as in childhood. Mostly congenital lesions constitute 20 to 32% of lesions located in the mediastinum. The main cystic masses are congenital benign cysts (bronchogenic, esophageal replications, neuroenteric, pericardial and thymic cysts), meningocele, mature cystic teratoma, and lymphangioma. In this study, we aimed to analyze the mediastinal cysts operated in our clinic according to the histopathological type, surgical type, morbidity rates and to contribute to the literature on these rare lesions.

**Methods** The records of patients with mediastinal cysts who were operated in Gazi University Faculty of Medicine Department of Thoracic Surgery, between January 2013 and June 2021, were reviewed retrospectively.

**Results** A total of 32 patients were included the study. Thirteen (40.6%) of the patients were male and 19 (59.4%) were female. The mean age was 45 (range: 12–71). The most common symptom in patients was chest pain with 12 patients. Histopathologically, the most common subtype was thymic cyst. Video-assisted thoracic surgery was applied in 19 patients (59.3%), thoracotomy in 8 patients (25%), and sternotomy in 5 patients (15.63%). There was no mortality.

**Conclusion** In patients with mediastinal cysts, the prognosis after complete excision is excellent and rates of morbidity and mortality associated with surgery are low.

Mediastinal cysts are well-margined, round, or oval lesions originating from the mediastinum compartments. These lesions develop as a result of cystic degeneration of a congenital, acquired, or solid tumor. Congenital lesions constitute 20 to 32% of the lesions in the mediastinum. 1 2 Mediastinal cysts are classified as congenital and acquired, according to the time of formation, and as foregut cysts, mesothelial cysts, lymphatic cysts, and thymic cysts, according to the tissue they originate from. The incidence of cysts may differ according to age. Foregut origin cysts are common in the neonatal period and childhood, while mesothelial cysts are more common in adulthood. In treatment, surgery has low mortality and morbidity rates and the surgical approach such as thoracotomy, cervical, transsternal, video-assisted thoracic surgery (VATS), or mediastinoscopy is determined depending on the location of the lesion. 2 3 In the present study, we aimed to analyze the mediastinal cysts operated on in our clinic according to the histopathological type, surgical type, and morbidity and recurrence rates and to contribute to the literature on these rare lesions.

## Materials and Methods

Following the approval of the local ethics committee (my institution does not have an Institutional Review Board), the records of mediastinal cyst patients who were operated on in Gazi University's Faculty of Medicine Department of Thoracic Surgery between January 2013 and June 2021 were reviewed retrospectively. We analyzed the patients' data in terms of age, sex, histopathology, lesion size, lesion localization, surgical technique, symptom, additional disease, length of hospital stays, and complications. However, the coronavirus disease 2019 pandemic has been continuing from March 2020 to the present. In this date range, emergency surgical interventions and especially malignancy surgery were performed. Since almost all of the mediastinal cystic lesions are benign and elective surgery is required for treatment, patients with mediastinal cystic lesions were not operated on within the specified date range.

## Statistics

The data were analyzed with SPSS (IBM, version 20, NY). The descriptive data were mean ± standard deviation, median (minimum–maximum), or number, and frequency. The chi-squared test was used for categorical variables and the log rank test was used for continuous variables.

## Results

A total of 32 patients were included in the study. The demographic and characteristic information of the patients is given in the table. Thirteen (40.6%) of the patients were male and 19 (59.4%) were female. The mean age was 45 (range: 12–71). The most common symptom was chest pain, in 12 patients. Histopathologically, thymic cyst ranked first, with 11 patients (34.40%). Pericardial cyst (15.62%) and bronchogenic cyst (15.62%) ranked second, with five patients each ( Table 1 ). Cystic teratoma in four patients (12.50%), Mullerian cyst in three patients (9.37%), lymphoepithelial cyst in two patients (6.25%), duplication cyst in one patient (3.12%), and gastroenteric cyst in one patient (3.12%) were also observed. In the distribution of lesion histopathology depending on age, in the pediatric group, consisting of one 12-year-old patient and one 17-year-old patient, a thymic cyst was seen in the 12-year-old patient and a gastroenteric cyst in the 17-year-old patient. There were seven cases in the 20 to 40 age group and the most common lesion was bronchogenic cysts in three patients. There were 23 cases in the group over age of 40 and the most common lesion was thymic cysts in 8 patients. The mean lesion size was 5.73 cm (range: 2–12). The lesions were located in the anterior mediastinum in 21 patients, in the posterior mediastinum in 6 patients, and in the middle mediastinum in 5 patients. When the patients were examined in terms of the surgical method, it was seen that VATS was applied in 19 patients (59.3%), thoracotomy in 8 patients (25%), and sternotomy in 5 patients (15.63%) ( Table 1 ). The mean length of stay was 4.53 days (standard deviation: 1.1). No postoperative complications were observed in 25 of the patients who were followed up. Four patients had wound infection, 2 patients had arrhythmia, and 1 patient had pneumonia. The morbidity rate was 21.9%. All of the patients who were followed up were discharged without any problem. There was no mortality.

**Table 1:** Epidemiological features and surgical results of our case series

		Study population *n* = 32	%
Gender			
	Male	13	40.6
	Female	19	59.4
Mean age	43.4		
Age range (y)	12–71		
Symptoms			
	Chest pain	12	
	Cough Asymptomatic	5 12	
	Shortness of breath Difficulty in swallowing	2 1	
Mean tumor diameter	5.73 cm		
Tumor diameter range (cm)	2–12		
Mean length of stay (d)	4.53		
Histopathological type	Thymic cyst Pericardial cyst Bronchogenic cyst Cystic teratoma Mullerian cyst Duplication cyst Lymphoepithelial cyst Gastroenteric cyst	11 5 5 4 3 1 2 1	
Postoperative complications	No complications Wound infection Arrhythmia Pneumonia	25 4 2 1	
The lesion location	Anterior mediastinum Posterior mediastinum Middle mediastinum	21 6 5	
Resection type			
	VATS	19	
	Thoracotomy Sternotomy	8 5	

Abbreviation: VATS, video-assisted thoracic surgery.

## Discussion

Congenital lesions constitute 20 to 32% of lesions in the mediastinum. 1 Although they are generally congenital, most cases are asymptomatic because the lesions are detected after the second decade. 2 The incidence of cysts may differ according to age. For example, although cysts originating from the foregut make up most of the neonatal and childhood cysts, they are rare in adults. Similarly, pericardial cysts comprise approximately one-third of mediastinal cysts seen in adulthood. 3 In our series, the age range was between 12 and 71 years and the mean age was 45 years. In the distribution of lesion histopathology depending on age, in the pediatric group, consisting of one 12-year-old patient and one 17-year-old patient, a thymic cyst was seen in the 12-year-old patient and a gastroenteric cyst in the 17-year-old patient. There were seven cases in the 20 to 40 age group and the most common lesion was bronchogenic cysts in 3 patients. There were 23 cases in the group over age of 40 and the most common lesion was thymic cysts in 8 patients. In the literature, it was reported that lesions were mostly seen in the pediatric age group and were generally congenital. 3 We consider that the reasons the patient group who was operated on in our series was mostly over the age of 40 are that the lesions do not reach symptomatic sizes in childhood, it is not considered as a priority in diagnosis due to the lesions not causing obvious symptoms, and parents avoid radiological imaging as they think that it may be detrimental to their children.

In general, all cysts have intrathoracic organ compression and related symptoms depending on their size. Some cysts are known to cause specific symptoms related to their structure, location, and formation mechanisms. 4 Our patients also had pressure on some mediastinal organs and related symptoms. Seven of our patients had trachea compression and associated cough and shortness of breath. In addition, one patient had difficulty in swallowing due to esophageal compression. Our 12 patients had chest pain due to heart and other mediastinal vascular organ compression, generally related to the lesion size. Depending on the compression of mediastinal organs, the surgical method has been changed in some cases. Conventional surgical methods were used for lesions with large size and organ compression. Gürsoy et al reported that patients were usually symptomatic (61%), chest pain was the most common symptom, and the other symptoms were dyspnea, cough, and hemoptysis, in descending order, in their series. 5 Bastos et al stated that the rate of symptomatic patients was 68% and the most common symptom was chest pain. 6 Similar to the literature, 62.5% of the patients were symptomatic in our series and the most common symptom was chest pain. Histopathologically, there are variable results for mediastinal cyst in the literature. In the study by Petkar et al, 39 mediastinal cysts surgically excised over 22 years were encountered, and histopathologically foregut cysts (19 cases, 50%) were the most common cysts, followed by teratomatous cysts (10 cases, 26.3%) and thymic cysts (4 cases, 10.5%). 7 Brzeziński et al reported that in their study of 50 patients histopathologically the most common cysts were bronchogenic cysts in 28 patients (56%), followed by pericardial cysts in 13 patients (26%), and enteric cysts in 8 patients (16%). 8 Jiang et al stated that 45 patients with mediastinal cysts underwent surgical treatment and histopathologically the most common cysts were bronchogenic cysts in 16 patients (35.5%), pericardial cysts in 14 patients (31%), and thymic cysts in 12 patients (26.5%). 9 In our study, histopathologically, thymic cyst was in first place, with 11 patients (34.40%). Pericardial cyst (15.62%) and bronchogenic cyst (15.62%) were in second place, with five patients each. Cystic teratoma in four patients (12.50%), Mullerian cyst in three patients (9.37%), lymphoepithelial cyst in two patients (6.25%), duplication cyst in one patient (3.12%), and gastroenteric cyst in one patient (3.12%) were also observed.

The general opinion is that the best treatment for mediastinal masses is complete resection, and the process that started with sternotomy and thoracotomy has historically evolved toward video-assisted surgery with the advancement in endoscopic imaging methods. In study by Aravena et al, it was reported that although transbronchial needle aspiration with endobronchial ultrasound (EBUS-TBNA) was indicated as a new method with a role in the treatment of bronchogenic cysts, EBUS-TBNA for mediastinal cysts was limited in diagnostic and therapeutic efficiency and surgical resection continued to be the preferred treatment. 10 In the study by Ulaş et al, VATS and thoracotomy results were compared in the treatment of mediastinal cystic lesions. Thoracotomy or VATS was applied in 60 mediastinal cyst patients. It was stated that the main treatment method for mediastinal cysts is surgery, and the thoracoscopic approach significantly reduces the patient's surgical procedure time and postoperative hospital stay. 11 In our series, VATS was applied in 19 patients (59.3%) and it provides adequate exposure for all anterior, visceral, and posterior mediastinal lesions. Thoracotomy was performed in eight patients and sternotomy in five patients. The general opinion of our clinic is to perform surgical total excision for cysts other than mesothelial cysts. The use of robotic surgery for mediastinal lesions has been increasing in recent years. 12 Due to the high cost of robotic surgery and the lack of surgical experience, the robotic surgery method was not used.

## Conclusion

In patients with mediastinal cysts, the prognosis after complete excision is excellent and rates of morbidity and mortality associated with surgery are low. However, for asymptomatic mediastinal cysts that do not compress the surrounding structures, expand gradually, do not show atypical features, or have low suspicion of malignancy, radiological follow-up and symptom follow-up may be recommended. It is very important that patients' follow-up and examinations are performed completely.
